# Randomized Controlled Trial of Durotomy as an Adjunct to Routine Decompressive Surgery for Dogs With Severe Acute Spinal Cord Injury

**DOI:** 10.1089/neur.2023.0129

**Published:** 2024-02-20

**Authors:** Nick D. Jeffery, John H. Rossmeisl, Tom R. Harcourt-Brown, Nicolas Granger, Daisuke Ito, Kari Foss, Damian Chase

**Affiliations:** ^1^ Department of Small Animal Clinical Sciences, Texas A&M University, College Station, Texas, USA.; ^2^ Department of Small Animal Clinical Sciences, VA-MD College of Veterinary Medicine, Blacksburg, Virginia, USA.; ^3^ Langford Vets, Bristol, United Kingdom.; ^4^ Bristol Vet Specialists, CVS Ltd, Bristol, United Kingdom.; ^5^ Nihon University College of Bioresource Sciences Department of Veterinary Medicine, Fujisawa, Japan.; ^6^ Department of Veterinary Clinical Medicine, University of Illinois Urbana–Champaign, Champaign, Illinois, USA.; ^7^ Veterinary Specialists Aotearora, Auckland, New Zealand.

**Keywords:** canine, disc herniation, dural incision, paraplegia

## Abstract

Although many interventions for acute spinal cord injury (SCI) appear promising in experimental models, translation directly from experimental animals to human patients is a large step that can be problematic. Acute SCI occurs frequently in companion dogs and may provide a model to ease translation. Recently, incision of the dura has been highlighted in both research animals and human patients as a means of reducing intraspinal pressure, with a view to improving perfusion of the injured tissue and enhancing functional recovery. Observational clinical data in humans and dogs support the notion that it may also improve functional outcome. Here, we report the results of a multi-center randomized controlled trial of durotomy as an adjunct to traditional decompressive surgery for treatment of severe thoracolumbar SCI caused by acute intervertebral disc herniation in dogs. Sample-size calculation was based on the proportion of dogs recovering ambulation improving from an expected 55% in the traditional surgery group to 70% in the durotomy group. Over a 3.5-year period, we enrolled 140 dogs, of which 128 had appropriate duration of follow-up. Overall, 65 (51%) dogs recovered ambulation. Recovery in the traditional decompression group was 35 of 62 (56%) dogs, and in the durotomy group 30 of 66 (45%) dogs, associated with an odds ratio of 0.643 (95% confidence interval: 0.320–1.292) and z-score of −1.24. This z-score indicates trial futility to reach the target 15% improvement over traditional surgery, and the trial was terminated at this stage. We conclude that durotomy is ineffective in improving functional outcome for severe acute thoracolumbar SCI in dogs. In the future, these data can be compared with similar data from clinical trials on duraplasty in human patients and will aid in determining the predictive validity of the “companion dog model” of acute SCI.

## Introduction

Poor functional recovery after severe spinal cord injury (SCI) has generated huge research effort from bench to bedside, but unfortunately the therapies available to reliably improve outcome remain limited, consisting primarily of decompressive surgery (DS) and physical therapy.^1–3^ Acute SCI alters tissue perfusion and activates a vast molecular cascade that culminates in progressive tissue loss.^3–7^ Impairment of the sodium-potassium exchange mechanism and changes in expression of aquaporin-4 lead to fluid accumulation within the spinal cord,^8–11^ which then can further hinder appropriate perfusion through an increase in intraspinal pressure (ISP).^12–14^

Although pharmaceuticals provide the most obvious treatment for acute SCI, there are many difficulties in delivering an active agent to the lesion within a short enough period after injury and at sufficient concentration to be effective in preserving injured tissue in clinical patients.^15,16^ One potential solution to circumvent the need for systemic delivery is to deliver the agent into the epidural space.^17,18^ A more prosaic approach that might potentially have broader therapeutic effect is to decrease the pressure within the dura, thereby increasing the effective perfusion pressure. Indeed, dural incision (durotomy) has been investigated frequently over many decades (over a century in total)^19^ as a means of potentially improving the outcome for affected persons.^20–22^ The effect of incising the meninges has been reinvestigated in more detail recently, using sensitive probes to define the effect on ISP. For instance, Khaing and colleagues demonstrated that incision of the dura reduces ISP and increases gray matter survival after acute contusion lesion in rats.^13,23^ The functional effect of a dural incision, with or without subsequent repair, has been less extensively investigated, with some reports in experimental animals suggesting improved locomotor outcome^24,25^ and some not.^26,27^

The recent resurgence of interest in durotomy has also been reflected in investigations in humans with SCI.^28^ These studies demonstrated reduction in ISP associated with duraplasty (repair of the incised dura is preferred in humans because watertight closure prevents leakage of cerebrospinal fluid [CSF] that can cause pain and/or neurological deficits). In a recent observational series, Phang and colleagues suggested that reduction in ISP associated with duraplasty was also associated with improved clinical outcome,^29^ providing the foundation for a clinical trial that is currently collecting data,^30,31^ and a management algorithm has been suggested for application of duraplasty in the clinic.^32^

Companion dogs frequently incur acute SCI of varying severity after acute disc herniation.^33^ Many small breeds of dog are chondrodystrophic, carrying an overexpressing FGF4 retrogene, which makes them susceptible to early-onset intervertebral disc degeneration and mineralization.^34–36^ Consequently, during a period of mechanical stress, affected dogs often rupture the annulus, thereby allowing the inner calcified nucleus to be forcibly expelled, creating a combined contusive/compressive spinal cord lesion.^37–39^ Although the SCI in many individuals is relatively mild, there is a subpopulation (∼20% of the total affected)^40^ with suprasacral lesions that lose all motor control and sensory function to the hindquarters (pelvic limbs, tail, bladder, and anus), and only ∼50% of such cases recover independent ambulation after conventional treatment (DS, rehabilitation, etc.).^41,42^ These dog patients have many similarities to American Spinal Injury Association Impairment Scale (AIS) human patients with grade A thoracic lesions,^43^ albeit with a much higher incidence of improvement than their human counterparts.^44,45^

The poor recovery rate for dogs in this subgroup has received considerable attention from veterinary neurosurgeons. The possible benefits of durotomy have been repeatedly investigated over many decades in experimental and clinical veterinary medicine, with discordant results.^19,46–48^ One obstacle to clear interpretation of the relevant veterinary clinical data is that, traditionally, observation of the spinal cord after durotomy was thought to allow diagnosis of myelomalacia, which in turn indicated a hopeless prognosis with consequent euthanasia of the affected dog.^49^ Unfortunately, this implies that retrospective observational studies^47,48^ are subject to substantial bias (because only the most severe-looking injuries receive durotomy). Durotomy as a potential treatment for severe SCI in dogs has been the subject of renewed interest during the past decade, with two prospective observational studies suggesting improved recovery of ambulation.^50,51^

It has frequently been suggested that this subset of spinal cord–injured dogs might form a suitable population for testing putative therapies that look promising in experimental SCI before they are tested in human patients.^52,53^ However, one stumbling block is that it is difficult to be sure how pertinent this model truly is in predicting the results of an outcome in similar human patients. The currently recruiting clinical trial of duraplasty in humans^30,31^ provides an opportunity for direct comparison with outcomes from the similar group of dogs that we report on here.

In this veterinary randomized controlled trial, we compared the proportion of companion dogs recovering to walk again after traditional DS for acute thoracolumbar disc herniation with that recovering after the addition of durotomy to the traditional approach.

## Methods

This was a randomized controlled trial to compare traditional surgery with traditional surgery plus durotomy in treatment of severe SCI caused by acute intervertebral disc herniation in dogs, approved by the Institutional Animal Care and Use Committee (IACUC 2019-0301CA; renewal: 2022-0191CA). This trial was designed with many pragmatic aspects because it enhances the generalizability of the results.^54^ As an example, for cases allocated to traditional surgery, the surgeon could decide to carry out any specific procedure and length of hemilaminectomy (as is routine in clinical practice) given that we were comparing “traditional surgery” against “new surgery.” The choice of perioperative medications was also at the discretion of the attending surgeon. Estimate of benefit associated with durotomy for sample-size calculations was based on a recent observational study.^50^

The dog spinal cord has a different relationship to the vertebral column than it does in humans: Dogs have 13 thoracic and seven lumbar vertebrae and the spinal cord terminates at around L6 vertebra (with slight variation with dog size). The pelvic limbs, bladder, and anus are innervated from lumbar segments L4–S3. Most acute disc herniation in dogs occurs in the vicinity of the thoracolumbar junction, and so the lesion creates upper motor neuron (i.e., suprasacral) deficits, although spinal shock is observed in some individuals for a short period of time.^55^

In veterinary medicine, acutely paraplegic dogs are first examined by general practitioners and then referred to specialist neurosurgical clinics for diagnosis and treatment, thereby inevitably incurring a delay of many hours before surgery is possible. At specialist clinics, affected dogs traditionally undergo cross-sectional imaging (computed tomography [CT] or magnetic resonance imaging [MRI]) and then are immediately taken for DS, usually by dorsolateral hemilaminectomy (the disc usually herniates to one side because of the midline dorsal longitudinal ligament) for removal of the herniated material from the epidural space. Post-operative care consists of standard nursing for the surgical site, bladder, bowel, and skin plus pain control using intravenous opioids for ∼48 h, followed by a variety of analgesic medications such as non-steroidal anti-inflammatory drugs (NSAIDs), acetaminophen, amantadine, codeine, etc.

### Study population

The study population was defined as dogs with severe acute thoracolumbar SCI caused by disc herniation. To be included, dogs must have had an absence of apparent conscious perception (i.e., turning to look, vocalizing, licking lips, etc.) of severe noxious stimuli (using smooth-surfaced pliers to create crushing injury) applied to the pelvic limb digits and tail. All included cases would as a matter of course be paraplegic and doubly incontinent. In terms of neurological deficits and severity, these cases resemble human thoracic AIS grade A patients.

Inclusion was dependent on MRI or CT localization of the herniated disc to within the T9–L3 region of the spinal cord. Dogs that had undergone surgical decompression for a previous thoracolumbar disc herniation were excluded, as were dogs with diabetes mellitus (because of association with worse outcome^56,57^), hyperadrenocorticism (association with muscle weakness, susceptibility to infection, poor healing, etc.), or those with severe concomitant disease (an expected life span of <9 months).

### Control

Control dogs underwent routine cross-sectional imaging (CT or MRI) to identify the herniated disc, followed by DS, usually hemilaminectomy of variable length, with removal of the herniated material from the epidural space.

### Intervention

The trial intervention was durotomy after routine cross-sectional imaging and removal of herniated disc material from the epidural space by standard DS as for control dogs. The length of the durotomy was set at four vertebral lengths, as a compromise between decompression over the full length of the swollen spinal cord as has been applied in human patients^29,30^ and the pragmatic need to limit surgical time to promote surgeon participation and was also used in a preliminary investigation of durotomy in dogs.^50^ Guidance about the extent of the durotomy was provided to contributing spinal surgeons, who were all experienced board-certified veterinary neurologist/neurosurgeons. The first incision in the dura was generally made with the bevel of a fine gauge (∼22-G) hypodermic needle and then enlarged using a number 12 blade, the bevel of a hypodermic needle, iris, or Castroviejo scissors or by tearing the edges with jewelers' forceps. The dura was left open, permitting free flow of CSF. In dogs, adverse effects of leaving the dura open are not detectable, perhaps because the vertebral column is held horizontally, and it is routine in veterinary surgery to leave such deficits open, with only rarely reported detrimental effects.^46–48^

### Outcome

In this study, commenced in October 2019, we defined recovery as the ability of a dog to take 10 consecutive steps in the pelvic limbs without falling within 6 months of the surgical intervention. (In dogs, recovery of ambulation after even very severe thoracolumbar spinal cord injury usually occurs within 3 months,^58^ but subsequent to durotomy we have recorded more prolonged recoveries in some individuals.^50^) The recovery of ambulation most often occurred after discharge from the clinic and was determined by in-person follow-up examination or documented by owners through video recordings sent to the investigators by e-mail or phone. At this stage of ambulatory recovery, most dogs also recover urinary and fecal continence, but this was not specifically recorded in this trial because recovery of these functions most often occurs after hospital discharge subsequent to this severity of SCI and owners often cannot distinguish between voluntary and involuntary voiding. Recovery of pain perception in the pelvic limbs was also not recorded, because of difficulties with owner interpretation or the need for dogs to be returned to clinics for veterinary evaluation.

It is recognized that dogs that have incurred severe thoracolumbar SCI have an approximate 10–15% likelihood of developing progressive myelomalacia that extends the region of spinal cord necrosis cranially, caudally, or both, causing pain and compromise of breathing, usually prompting euthanasia.^59,60^ This form of progressive spinal cord destruction occurs within ∼10 days of the inciting injury and was also recorded for dogs in this study.

### Sample-size calculation

Many decades' data have demonstrated that, subsequent to traditional decompressive spinal surgery, the recovery rate for dogs that have lost pain perception in the pelvic limbs is ∼55%.^41,42^ A recent observational study on durotomy added to routine DS suggested a recovery rate of ∼72% (95% confidence interval [CI]: 52–87),^50^ and therefore the required sample size was calculated based on the ability of durotomy to improve the recovery rate from 55% to 70%. We reasoned that the extra time required for durotomy would not be considered worthwhile unless there was an improvement of at least this magnitude. Sample size was calculated based on a power of 80% and alpha of 0.05, resulting in a calculated total study population of 326, randomized 1:1 between traditional surgery and 4-vertebral-length durotomy. Allowing for a loss to follow-up of ∼10%, we aimed to recruit 360 cases in total. This number of cases cannot be collected within a reasonable time by a single veterinary clinic, and so this was a multi-center study.

### Randomization

After a suitable case was identified by the attending clinician, the owner was informed about the trial and its purpose and was asked to provide informed consent. For those cases that were enrolled, the treatment allocation was achieved through opening a pre-prepared opaque envelope containing either “‘traditional”’ or “‘durotomy”’ instruction. The case then received the allocated treatment. Deviations from protocol were recorded, but dogs were analyzed according to the treatment they actually received. Dogs at each contributing center were randomized independently of other centers.

### Data collection and analysis

Outcome data were initially collected onto specifically designed Case Report Forms, detailing demographics, duration of clinical signs (e.g., back pain) before onset of paralysis (interval 1), previous prescription (by the referring general practice veterinarian) of medications, interval between onset of paralysis and presentation to the surgical clinic (interval 2), and intraclinic interval till completion of surgery (interval 3). The site and location of the disc herniation and the length (measured in vertebral body lengths) of the ablation of visible subarachnoid fluid on MR myelographic (highly T2-weighted) images^61^ were recorded when available (dogs did not have to undergo MRI to be included). These Case Report Form data were then sent to the central coordinator for entry into a spreadsheet.

### Statistical analysis

Demographic data were summarized by mean and standard deviation for normally distributed data and by median and interquartile range (IQR) for non-normally distributed data. We planned to use logistic regression to calculate the odds ratio of recovery in the two groups to determine the effectiveness of the durotomy, assuming *p* < 0.05 as significant. The intention was to collect 360 cases, but, because recruitment in this trial was adversely affected by the SARS-CoV2 pandemic and the recruitment rate did not recover afterward, interim analyses with conservative stopping rules were implemented at one third of the proposed total recruitment, with intention to continue with subsequent analyses at 67% and 100% of the total study population, if appropriate. We used the O'Brien-Fleming stopping rule^62^ because of its conservative nature (more likely to continue the trial to the original end-point). Although the weight of evidence favors early DS after SCI, there is still some controversy around timing in both humans and dogs,^63–69^ so we also included exploratory analysis of recovery of ambulation and interaction between durotomy and time interval from presentation to the surgical clinic and completion of surgery (the only time interval that is under control of the surgeon).

Stata 18 (StataCorp, College Station, TX) was used for all summary statistics, sample-size calculation, and data analysis.

## Results

**FIG. 1.**
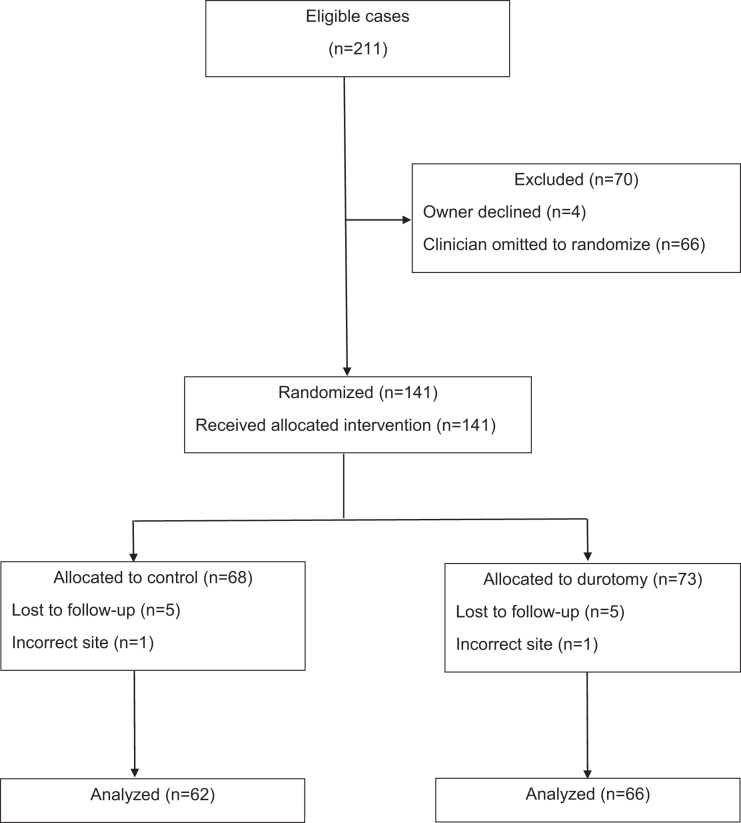
CONSORT diagram illustrating the flow of eligible cases through this parallel arm randomized trial.

At 3.5 years after commencing the trial, we had enrolled 141 dogs, of which 128 had appropriate follow-up (2 cases were excluded because the disc herniation occurred at L4/5 [outside the study region] and 11 cases [6 durotomy; 5 traditional] were lost to follow-up; Fig. 1). Fifty-two of the 128 (41%) dogs were dachshunds of various types (the dachshund is a breed well known to be susceptible to this type of acute disc herniation).^33–36^ As expected in a randomized data set, the various recorded demographic characteristics and pre-operative variables were balanced between groups (Table 1).

**Table 1.** Demographic and Clinical Presenting Information on the Study Population

	***Traditional*** (**n* = 62)***	***Durotomy*** (**n* = 66)***	***Total*** (**n* = 128)***
Age (years, IQR)	5 (4–6)	4 (3–6)	5 (3–6)
			
Weight (kg, IQR)	7.6 (5.5–12.9)	7.8 (6.0–11.6)	7.8 (5.8–11.8)
			
Sex			
Female/desexed	31 (51%)/29	36 (55%)/29	67 (53%)/58
Male/desexed	31/23	30/24	61/47
			
Dachshund	24 (38%)	28 (42%)	52 (41%)
			
Reflexes			
Reduced	33 (53%)	36 (55%)	69 (54%)
Normal	29	30	59
			
CSF “gap” ^ (IQR)	5 (3–7)	5.5 (4–7)	5 (3.5–7.0)
	(*n* = 44)	(*n* = 51)	(*n* = 95)
			
Pre-operative medication			
None	24	25	49
NSAID	21	30	51
Glucocorticoid	17	11	28
			
Site			
T10	0	2	2
T11	7	10	17
T12	18	11	29
T13	14	19	33
L1	8	8	16
L2	9	6	15
L3	6	10	16
			
Interval 1 (h, IQR)	16 (4–72)	24 (8–36)	21.5 (5–48)
			
Interval 2 (h, IQR)	18 (8–48)	17.5 (8.5–44.0)	18 (8–44)
			
Interval 3 (h, IQR)	7 (5–13)	6.5 (5–10)	7 (5–12)

Key: ^ CSF “gap” = length of interruption of visible fluid in subarachnoid space on heavily T2-weighted MR images, measured in vertebral body lengths. Interval 1 = time between first thoracolumbar clinical signs and inability to walk. Interval 2 = time between when first unable to walk and presentation to the surgical clinic. Interval 3 = time between presentation at the surgical clinic and completion of surgery.

IQR, interquartile range; CSF, cerebrospinal fluid; NSAID, non-steroidal anti-inflammatory drug.

Of the 128 included cases, 65 (51%) recovered ambulation and 63 did not. In the durotomy group, 30 of 66 (45%; 95% CI: 34–57) dogs recovered, compared with 35 recoveries in 62 dogs (56%; 95% CI: 43–69) undergoing traditional DS alone. Both these proportions are within the ranges previously reported for dogs that have lost pain perception in their hindquarters.^41,42^ Logistic regression indicated an unadjusted odds ratio for recovery for durotomy versus traditional surgery alone of 0.643 (95% CI: 0.320–1.292) and z-score of −1.24.

At this stage, because we had recruited one third of our projected sample and recruitment was slowing owing to the disruption of clinical services during and after the SARS-CoV2 pandemic, we carried out an interim analysis. The raw data indicated that durotomy was associated with slightly worse outcomes than traditional surgery and so the analysis focused on futility. The z-score for unadjusted logistic regression was −1.24, the negative value indicating reduced likelihood of recovery for cases undergoing durotomy. This value is less than the z-score futility bound of −0.025 for a population size of 122 (non-binding O'Brien-Fleming rule^62^; see Supplementary Table S1, Supplementary Figure S1, and Supplementary Commentary), indicating that the data collected to this point were incompatible with the target beneficial effect of durotomy of ≥15% above traditional surgery, and so the trial was terminated at this stage.

The exploratory analysis of interaction between durotomy and the time interval from presentation to the surgical clinic and completion of surgery suggested that delay in completing surgery after presentation to the surgical clinic is overall associated with a detrimental effect (odds ratio [OR]: 0.905; 95% CI: 0.842–0.974; *p* = 0.007). However, there was a non-significant interaction between interval to completion of surgery and type of surgery (OR: 1.016; 95% CI: 0.879–1.174; *p* = 0.833; i.e., the timing of surgery does not affect the relative efficacy of durotomy vs. traditional decompression). Of the 128 dogs included, 10 (8%) developed progressive myelomalacia and were euthanatized, 4 in the durotomy group and 6 in the traditional surgery group.

## Discussion

The proportion of recovered dogs in this population (51%) is similar, albeit slightly lower than that typically reported and, despite previous observational data suggesting that durotomy was associated with increased recovery after acute thoracolumbar SCI in dogs,^50,51^ this randomized trial provides strong evidence that it is ineffective and even hints at the possibility of negative effects. Perhaps the most likely explanation for the discrepancy between this trial and previous reports is the observation that summary outcomes are often more extreme when analyzing small samples, especially observational data, whereas the underlying true effect of durotomy in this patient population is null (as detected in this more rigorous controlled trial).

In experimental animals, the effect of durotomy on intraparenchymal pressure is not large; the reduction is an average of 2.2 mm Hg (∼25% of the pressure before intervention) during the first 24-h period,^13^ and it could be considered that this may not be sufficiently effective in restoring blood flow that is compromised because of raised intradural pressure. Further, Prasse and colleagues^70^ provide evidence to suggest that although durotomy may improve functional outcome after more moderate contusive injuries, this effect is not detectable in severe SCI. From a mechanistic point of view, it must also be considered that the compromise in blood flow associated with SCI is not solely a result of raised intraparenchymal pressure, but also a consequence of the numerous biochemical cascades that compromise both blood vessel integrity and intraluminal cross-sectional area.^4,71,72^ For instance, release of agents such as ED1 and hemoglobin causes vasoconstriction,^73–75^ and upregulation of sulfonylurea receptor 1 induces damage to endothelial cells, predisposing to both hemorrhage and thrombosis,^76^ with a combinatory effect of reducing blood flow that cannot be reversed solely by eliminating the constricting effect of the dura.

It has frequently been asserted that SCI in dogs provides a clinically relevant model in which to assess the impact of various therapeutic interventions before embarking on the expense of a clinical trial in humans.^52,53^ The results of this study in dogs can be regarded as strongly suggesting that dural incision in human patients will not be effective in promoting restoration of function. However, the currently recruiting clinical trial on duraplasty for severe SCI in humans^30,31^ has several potentially important differences from this current dog trial that may impact on outcome. Most notably, the human trial focuses on cervical lesions (rather than thoracolumbar in the dogs), is testing duraplasty rather than durotomy, and the length of durotomy varies between patients (and perhaps a case-specific durotomy length could have been more beneficial in dogs). An unclosed durotomy, as in this dog trial, may promote adhesions between the pial surface and external tissue, as has been detected in some experimental models,^25,77^ and might have a deleterious effect on functional recovery. This risk should be largely avoided in human patients who routinely undergo duraplasty.

Nevertheless, unclosed durotomy is customary in dogs, and, although late complications have occasionally been noted after dural incisions for congenital spinal cord lesions,^78^ evidence for a systematic detrimental effect on functional outcome after SCI in dogs has not previously been suspected.^46–48^ There is also a need to recognize that the contusive/compressive lesion resulting from acute disc herniation in dogs does differ from SCIs caused by external trauma, as is more common in humans, most notably because of the association with systemic shock and multi-system injury. In summary, there are some differences in detail between the trials in the two species, which may weaken the predictive ability of this dog trial for outcomes in humans. It will be instructive to compare the outcomes of the current human trial with the null effect for dural incision forecasted by this dog trial of durotomy because it is plausible that, despite differences in detail of technique and case selection, this “companion dog model” of SCI may still have predictive validity.

Our exploratory analysis supports the concept that “time is spine”^79^—that is, that delay to DS may be associated with poorer outcomes, as has been suggested in many, but not all, analyses of observational data in human SCI. However, the reliability of this aspect of our analysis is uncertain because the study was not powered to detect this effect. In veterinary medicine, it has been suggested that durotomy may also reduce the likelihood of development of progressive myelomalacia in dogs after severe disc-associated SCI.^80–82^ However, because of the low incidence of this condition even in dogs with loss of pain perception in their hindquarters, an adequately powered study requires many hundreds of cases to be definitive and we cannot draw conclusions from the low number of affected dogs (*n* = 10) in this data set.

Many dogs that were eligible for this comparative study were not randomized (Fig. 1), and it must be considered whether their non-enrollment might affect the outcome data we record here. The reason for non-enrollment was largely because owners declined or, more commonly, clinicians forgot to mention the trial to the owners, and this became more common as the trial continued, owing at least partly to disruption associated with the SARS-CoV2 pandemic. Although it is not optimal that so many cases were not enrolled, their non-inclusion was not systematic and would therefore appear unlikely to affect our central conclusion.

Ideally, in this type of trial, it would be helpful to also record recovery of pain perception in the pelvic limbs plus recovery of fecal and urinary continence. We did not aim to collect this information in this trial because these functions recover after dogs have been discharged from the hospital and owners cannot accurately record any of these outcomes, necessitating return to the clinic for assessment at regular intervals. Without substantial financial incentives, owner compliance for regular reassessment is poor and so we focused on ambulation, which can be unequivocally assessed without in-person examination. As a consequence, it is possible that some of the dogs that are categorized as recovering ambulation may be so-called spinal walkers, that is, dogs that are walking without clinical evidence of communication between the brain and pelvic limbs.^83^ This may lead to misestimation of the incidence of “true” recovery in our results, although such an effect is likely to be minimal because recovery of spinal walking to walk 10 steps without falling is uncommon and the incidence of recovered ambulation in this trial is similar to that reported in other studies.^41,42^

Further, the key study aim was to compare outcomes between traditional DS and durotomy, and there is no reason to suppose that there will be a differential incidence of spinal walking between these treatment groups.

We deliberately chose not to standardize the surgical DS in this trial because the question asked in this trial is whether durotomy adds benefit to traditional decompression as selected by an experienced veterinary neurosurgeon, and also because it promotes generalizability of our results. It is possible that this lack of standardization may have altered the calculated difference (odds ratio) in outcome between techniques (because some of the traditional surgery decisions may have been superior to others), but does still support a generalizable conclusion that, overall, durotomy is not superior to conventional DS.

## Conclusion

This trial strongly suggests that durotomy is not beneficial in improving the outcome after severe acute thoracolumbar SCI in dogs. If our study correctly predicts that duraplasty is similarly ineffective in the current trial in human cervical SCI,^30^ it will provide strong evidence to support the notion that SCI in companion dogs also has predictive validity as a model in which to test other putative therapies before embarking on expensive human clinical trials.

## Supplementary Material

Supplemental data

Supplemental data

Supplemental data

## Supplementary Material

Supplementary Figure S1

Supplementary Commentary

## Abbreviations Used

AIS: American Spinal Injury Association Impairment Scale
CI: confidence interval
CSF: cerebrospinal fluid
CT: computed tomography
DS: decompressive surgery
ISP: intraspinal pressure
MRI: magnetic resonance imaging
NSAIDs: non-steroidal anti-inflammatory drugs
OR: odds ratio
SCI: spinal cord injury
