# Supplementary material for: Randomized Controlled Trial of Durotomy as an Adjunct to Routine Decompressive Surgery for Dogs With Severe Acute Spinal Cord Injury
Source: Neurotrauma Rep. 2024 Feb 20;5(1):128–38. doi: 10.1089/neur.2023.0129 (PMC10898236; doi:10.1089/neur.2023.0129)
Supplement: Supplemental data [file Suppl_TableS1.docx]

**Analysis for stopping decision**

**1. Supplemental table:** Results of O’Brien-Fleming nonbinding one-sided two sample proportions test for futility, using an expected baseline of 55% recovery and improvement to 70% associated with durotomy.

| Look | Info.prop | Futility bound  z-score | Sample size  N1 N2 N3 | | |
| --- | --- | --- | --- | --- | --- |
| 1 | 0.33 | -0.0249 | 61 | 61 | 122 |
| 2 | 0.67 | 1.194 | 121 | 121 | 242 |
| 3 | 1 | 1.96 | 182 | 182 | 364 |

**Key:** **Info.prop** – information proportion.
