# Supplementary material for: Randomized Controlled Trial of Durotomy as an Adjunct to Routine Decompressive Surgery for Dogs With Severe Acute Spinal Cord Injury
Source: Neurotrauma Rep. 2024 Feb 20;5(1):128–38. doi: 10.1089/neur.2023.0129 (PMC10898236; doi:10.1089/neur.2023.0129)
Supplement: Supplemental data [file Suppl_FigS1.docx]

**2. Supplemental Graph:** Analysis in the table shown in graphic format
