# Supplementary material for: Randomized Controlled Trial of Durotomy as an Adjunct to Routine Decompressive Surgery for Dogs With Severe Acute Spinal Cord Injury
Source: Neurotrauma Rep. 2024 Feb 20;5(1):128–38. doi: 10.1089/neur.2023.0129 (PMC10898236; doi:10.1089/neur.2023.0129)
Supplement: Supplemental data [file Suppl_Commentary.docx]

**Commentary**

At the first look, when 33% (*i.e.* 61 dogs in each group) of the projected study population have been recruited and followed up, the z-score to avoid stopping for futility is -0.0249.

The interim analysis when we had recruited ~33% of the population (62 traditional/66 durotomy) found a considerably lower z-score (-1.24), suggesting that durotomy was insufficiently effective to reach the 15% increase in absolute recovery rate if the study were to be continued. We therefore decided to terminate the study for futility.
